# PbSe/PbS Core/Shell Nanoplatelets with Enhanced Stability and Photoelectric Properties

**DOI:** 10.3390/nano13233051

**Published:** 2023-11-29

**Authors:** Anton A. Babaev, Ivan D. Skurlov, Sergei A. Cherevkov, Peter S. Parfenov, Mikhail A. Baranov, Natalya K. Kuzmenko, Aleksandra V. Koroleva, Evgeniy V. Zhizhin, Anatoly V. Fedorov

**Affiliations:** 1 PhysNano Department, ITMO University, Saint Petersburg 197101, Russia; 2 Research Center for Optical Materials Science, ITMO University, Saint Petersburg 197101, Russia; 3 Research Park, St. Petersburg State University, Saint Petersburg 199034, Russia

**Keywords:** 2D materials, photoconductivity, photoconductor, lead selenide, cation exchange

## Abstract

Lead chalcogenide nanoplatelets (NPLs) have emerged as a promising material for devices operating in the near IR and IR spectrum region. Here, we first apply the cation exchange method to PbSe/PbS core/shell NPL synthesis. The shell growth enhances NPL colloidal and environmental stability, and passivates surface trap states, preserving the main core physical properties. To prove the great potential for optoelectrical applications, we fabricate a photoconductor using PbSe/PbS NPLs. The device demonstrates enhanced conductivity and responsivity with fast rise and fall times, resulting in a 13 kHz bandwidth. The carrier transport was investigated with the field effect transistor method, showing p-type conductivity with charge mobility of 1.26 × 10^−2^ cm^2^·V^−1^·s^−1^.

## 1. Introduction

New-generation devices require novel materials with tunable physical properties and processing suitable for low-cost and low-temperature fabrication. 0D semiconductor nanocrystals (NC), also referred to as quantum dots (QDs), were among the first materials that fulfill these criteria, attracting significant attention from the field of nanotechnology [1]. Recently, the Nobel prize in chemistry was awarded for their development [2]. One of the most important spectral ranges in which QD properties could be useful is near-infrared (NIR). Biological tissue and telecommunication fiber transparency windows lay in the NIR and require high-quality light emission sources and detectors. On the other hand, effective and conductive light absorption materials are needed for efficient solar energy harvesting. The most promising compounds for NIR NC development are lead chalcogenides. Lead chalcogenide QDs have been extensively studied for almost two last decades and still appear to be an auspicious material for NIR applications [3,4,5,6]. The great progress on lead chalcogenide QDs has generated an astonishing toolkit for NC physical properties modification for optoelectronics applications, namely, control stoichiometry, size, crystal orientation, geometrical form and surface morphology of the NCs, dopant and ligand engineering and heterostructures fabrication [7]. However, the fundamental limitation of 0D NC impeding their application still exists, namely, low conductivity resulting from the abundance of surface defects [8]. The other problem with the PbS-based detectors could be their low bandwidth [9], while PbSe QDs suffer from low environmental stability [10].

To overcome these limitations, more complex NC shapes like 1D or 2D could be applied [11]. The poor conductivity problem of QD-based films arises from the atom-like energy level structure, which is a consequence of a 3D quantum confinement. QD-to-QD charge transfer usually has a hopping nature because of the QDs’ size distribution, resulting in low values of both conductivity and charge carriers’ mobility (typically 10^−3^ cm^2^·V^−1^·s^−1^) [12,13]. To achieve band-like charge transfer in QD solids, it is required to form the QD superlattice, which is challenging to produce for large-area devices. In contrast, 1D quantum confinement in nanoplatelets (NPLs) results in a continuous energy spectrum of conduction and valence bands, thus solving that problem [14].

The research on 1D confined lead chalcogenide colloidal NC started in 2010 when Schliehe et. al. [15] published the first protocol for PbS nanosheets synthesis. Since then a variety of concepts and approaches has been developed [11]. The recent work of Galle [16] highlights PbSe NPLs as a perspective material for NIR applications. The bare NPLs-based photoconductor shows fast response unreachable in PbS QDs-based counterparts. However, the NPLs still have low stability and the overall device performance is far from optimal.

Here, we report on a new step in lead chalcogenide 2D nanocrystals development. We have provided a three-step synthesis to obtain PbSe/PbS core/shell NPLs. The Pb cation exchange kinetics has been analyzed using aliquoted synthesis. The resulting NPLs possess good stability in ambient air conditions. As proof of this concept, we fabricate a photoconductor device using synthesized NPLs with different surface ligands. The photoconductors fabricated show promising responsivity from NIR to visible spectral ranges, good environmental stability, and bandwidth above 10 kHz.

## 2. Materials and Methods

### 2.1. Chemicals

Cadmium oxide (CdO), myristic acid (HMyr), cadmium acetate dihydrate (Cd(OAc)_2_ × 2H_2_O), 1-octadecene (ODE), oleic acid (OA), oleylamine (OlAm), N-methylformamide (NMF), ammonium sulfide solution ((NH_4_)_2_S), selenium oxide (SeO_2_), selenium powder (Se), lead bromide (PbBr_2_), isopropyl alcohol (IPA), toluene, acetonitrile, tetrachloroethylene (TCE), 1,2 ethanedithiol (EDT), and tetrabutylammonium iodide (TBAI) were purchased from Sigma Aldrich and used without further purification.

### 2.2. Synthesis of CdSe NPLs

CdSe NPLs with a 4 monolayer (ML) thickness were synthesized following the method developed by Galle et al. [17]. Briefly, a mixture of CdO (70 mg) and HMyr (340 mg) in 15 mL of ODE was heated in a flask at 180 °C for 30 min under Ar+ conditions until colorless. In the next step, Se powder (24 mg) was dispersed in 3 mL ODE by ultra-sonication for 30 min. This dispersion and 10 mL of ODE were added to the cooled flask with Cd-precursor at 100 °C, followed by a degassing step. Then, the flask was filled with argon and the temperature was raised to 240 °C. Upon heating, Cd(OAc)_2_ × 2H_2_O (160 mg) was swiftly added once the solution became orange (near 210 °C). Upon reaching 240 °C, the solution of SeO_2_ (40 mg) in 5 mL ODE (prepared by heating the mixture to 200 °C until a clear orange solution formed) was injected at a rate of 25 mL/h (10 min). An additional selenium-precursor was injected during the growth period to increase the lateral size of the NPLs. The NPLs were left to grow for 30 min and thereafter the flask was cooled down. At 165 °C, 2 mL of OA, and at 80 °C, 20 mL of IPA were added with subsequent centrifugation. The precipitated NPLs were dispersed in 4 mL of n-hexane.

### 2.3. CdS Shell Growth on the CdSe NPLs

A layer-by-layer process was used for the CdS shell growth. An amount of 1 mL of an initial solution of CdSe NPLs in hexane and 2 mL of IPA was added and into a 15 mL centrifuge tube and subsequently centrifuged. After the precipitation NPLs were dispersed in 3 mL of toluene followed by and addition of 3 mL of N-methylformamide (NMF), thus forming two-phase solution, 50 μL of (NH_4_)_2_S solution were added to the mixture and the centrifuge tube was stirred for 5 min. As the exchange of ligands at the surface occurred, the NPLs’ color turned red and NPLs transferred from the toluene to the NMF. The resulting solution was centrifuged with 3 mL of toluene and 1.5 mL of acetonitrile. The precipitate was dispersed in 2 mL of NMF. Then, 1 mL of a 0.3 M solution of (Cd(OAc)_2_·2H_2_O) in NMF was added and the solution was stirred for 5 min. The resulting CdSe NPLs with one layer of CdS shell were precipitated with toluene and acetonitrile mixture (1:1 vol) and redispersed in 2 mL of toluene with an addition of oleylamine (0.2 mL).

### 2.4. Cation Exchange

A mixture of PbBr_2_ (32 mg), 1 mL OlAm, and 4 mL ODE was degassed at 100 °C for 30 min. The temperature was lowered to 80 °C and the flask was filled with argon. At this temperature, 1 mL of CdSe/CdS core/shell NPLs solution (diluted by 2 times with toluene) were injected into the flask. The reaction proceeded at 80 °C for 3.5 h. After that, the PbSe/PbS NPLs were precipitated by centrifugation with the mixture of toluene (3 mL), IPA (12 mL) and OA (0.5 mL). Precipitated NPLs were dispersed in TCE.

### 2.5. Methods

Absorption spectra were measured using a Shimadzu UV3600 spectrophotometer (Shimadzu corporation, Kyoto, Japan). Photoluminescence (PL) spectra and PL decay curves in the NIR were measured using a custom-built setup [18]. For the PL decay measurements, a 532 nm Nd:YAG laser (STANDA, Vilnius, Lithuania) with a 1 ns pulse duration was used for excitation, and an InGaAs/InP avalanche photodiode (APD) operating in a photon-counting mode was used for light detection. For the PL spectra measurements, a 633 nm He-Ne laser was used for excitation, and an InGaAs photodiode was used for detection. The scanning electron microscopy (SEM) measurements were performed using a Merlin Zeiss electron microscope (Carl Zeiss, Oberkochen, Germany) in high vacuum mode at 10 kV accelerating voltages. I-V measurements were taken using a Keithley 2636b Source Measure Unit (Keithley Instruments, Solon, OH, USA). For the rise and fall times measurements we combined a high-speed current amplifier HCA-10M-100K-C (FEMTO Messtechnik, Berlin, Germany) with a Tektronix TDS2022B (Tektronix, Beaverton, OR, USA) oscillograph with the abovementioned 532 nm Nd:YAG laser used for the excitation. X-ray photoelectron spectroscopy (XPS) measurements were performed on an Escalab 250Xi photoelectron spectrometer (Thermo Fisher Scientific, Waltham, MA, USA) equipped with an AlKα monochromatic radiation source (photon energy 1486.6 eV). The spectrometer was calibrated against the Au 4 f7/2 line (binding energy 84.0 eV). The spectra were recorded in the constant transmission energy mode at 50 eV with an XPS spot size of 650 μm. The total energy resolution of the experiment was about 0.3 eV. The studies were carried out at room temperature in an ultrahigh vacuum of the order of 1–10^−9^ mbar. To remove the sample charge, a combined ion-electronic charge compensation system was used. The X-ray diffraction analysis (XRD) was performed on an Ultima IV (Rigaku, Tokyo, Japan) diffractometer with Cu Kα radiation (λ = 1.5418 Å).

## 3. Results and Discussion

### 3.1. The Optical Properties Evolution during Synthesis

To better understand the structure evolution during the exchange process, we performed absorption and PL measurement of aliquots taken during the exchange reaction. The distinguished parent CdSe/CdS NPLs heavy-hole (2.1 eV) and light hole (2.27 eV) excitonic absorption peaks progressively diminish during the reaction and disappear almost completely only after 240 min (Figure S1a) of reaction time. It is also worth noting that PbSe/PbS NPL fundamental transition redshifts for about 100 meV. This shift occurs only during the first 180 min of the reaction and then ceases (Figure S1b). We attribute this to the initial fast exchange reaction followed by slow guest ions diffusion, which has been reported before for the core-type PbSe NPLs synthesis [17].

**Figure 1:**
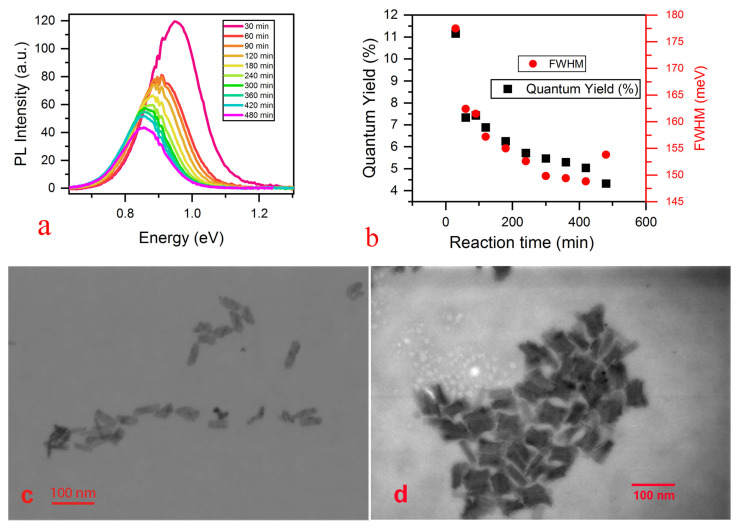
(**a**) PL spectra of the PbSe/PbS core-shell NPLs during cation exchange reaction, (**b**) corresponding QY and FWHM values; STEM images of initial CdSe/CdS (**c**) and resulting PbSe/PbS (**d**) NPLs.

Contrary to the absorption, PL demonstrates continuous evolution throughout the exchange reaction. The PL peak corresponding to the PbSe/PbS NPLs emission gradually redshifts from 0.94 to 0.85 eV (Figure 1a). Thus, the NPLs stokes shift increases after 180 min reaction time. Salzmann et. al. also observed a similar phenomenon for the PbSe core-type NPLs cation exchanged from CdSe and explained it via the continuous growth of the PbSe domain within the NPL [19]. Additionally, the aforementioned paper describes the possible appearance of heterojunctions between CdSe and PbSe clusters, which could also have an impact on optical properties. Both PLQY and the FWHM of the NIR band decreases during the reaction (Figure 1b). The prolonged reaction also initiates the structural degradation of the NPLs, which results in PL spectra widening that appears after 420 min of the reaction. The detailed study of the reaction kinetics lies beyond the scope of this paper. The optimal reaction time was found to be 3.5 h; it was used to obtain the NPLs for detailed characterization later on.

The morphology of the NPLs suffers no significant change during the exchange reaction. The STEM images (Figure 1c,d) show the rectangle shaped NPLs with 15–20 to 50 nm lateral sizes and thickness of about 2 nm. The NPLs tend to stack; however, the solution can be easily filtered from the large aggregates using a 0.22 µm PTFE syringe filter (Ossila, Sheffield, UK) for the ink preparation.

**Figure 2:**
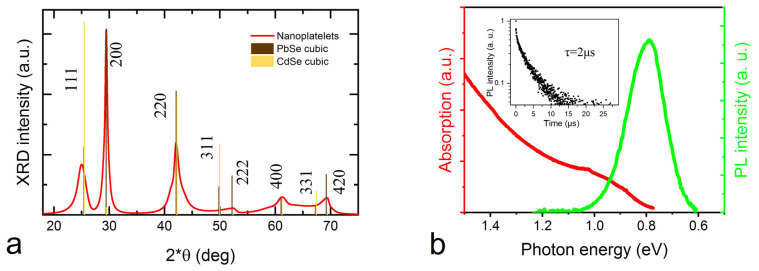
(**a**)—X-ray diffraction pattern of the PbSe/PbS NPLs (red) with crystal planes diffraction peak positions and intensities for cubic PbSe (brown) and cubic CdSe (yellow) lattices. (**b**)—PbSe/PbS NPLs absorption and PL spectra, inset—PL decay curve.

The X-ray diffraction analysis (Figure 2a) suggests that synthesized PbSe/PbS nanoplatelets have predominantly a cubic rock-salt PbSe structure, much like the core-only PbSe NPLs [17]. We can confirm the change in the crystal lattice from CdSe cubic rock salt to PbSe cubic rock salt due to the change in the [111] to [200] peak intensities. Interestingly, we observe increased intensity of the diffraction peak corresponding to the [200] PbSe plane (29.4°). We can speculate that the reason for this is the NPLs’ preferred orientation along the [200] plane [20].

The elemental analysis of the sample was performed using EDX measurements (Figure S2, Table S1) and X-ray photoelectron spectroscopy (see Figures S3 and S4 and Table S2). We observe about 10–18% of residual Cd atoms relative to the Pb atoms. The presence of residual host atoms is common for cation exchange because of low Pb^2+^ ions diffusivity coupled with the absence of preferred entry points in the cubic structure [21]. In both XPS and EDX data we observe bromide signal, which appears to be a lead bromide layer on the NPLs surface, resulting from the excessive amount of PbBr_2_ in the reaction solution. The exact ratio of anions to Pb suggests both X and Z types passivating of the NPLs. The presence of Br and residual Cd was noted in the pioneering work at the CdSe to PbSe NPLs cation exchange [17]. Comparing the obtained diffraction pattern to the bulk PbSe standard, we can also observe the 0.4° shift of the [111] crystal plane to the lower angles. This phenomenon originates from the appearance of an extra diffraction peak between the forbidden [110] and allowed [111] diffraction peaks, thus appearing as a shift of the [111] plane peak. This extra diffraction peak originates from the pair interactions between the same atoms (Pb-Pb, Se-Se, residual Cd-Cd and perhaps even Pb-Cd) in different monolayers of the NPL. The similar behavior in the diffraction patterns of CdSe NPLs was explained in detail in a paper by Chen et al. [22].

The PL spectra of the PbSe/PbS NPLs can vary from batch to batch, mostly due to the poor control of the parent CdSe/CdS NPLs lateral sizes. PbSe has the Bohr exciton radius of 46 nm [23] which results in a quasi-3D quantum confinement in PbSe/PbS NPLs. Average PbSe/PbS NPLs have the PL peak position at 1450–1550 nm with FWHM of 160–170 nm and PLQY of ~0.13. The colloidal solutions are rather stable even after 25 days of storage in ambient air conditions: the PL band redshifts for 12 nm with no FWHM change (Figure S5). Compared with the PbSe core-type NPLs [17] with the same solvent (TCE), the stability is enhanced.

PL decay curves for the PbSe/PbS NPLs are best fitted with a three-exponential decay function. Such a decay curve shape for PbSe NPLs has been observed before [24], but the attribution of the individual decay components still remains unclear. The obtained PbSe/PbS NPLs demonstrate PL lifetimes of about 2 µs, which is relatively long compared with the NIR-emitting PbSe QDs [25], as well as core-only PbSe NPLs [24]. Longer PL decay times for the core-shell NC compared with the core-only NC is a well-known phenomenon and is usually attributed to the increase of a radiative decay constant [26]. Interestingly, it has been shown that PbSe NPLs PL lifetimes increase with NPLs thickness and can vary from 0.7 μs to 2.7 μs for thicknesses 2–6 mL, respectively [17]. It is worth noting that our PbSe/PbS NPLs with low PLQY demonstrate shorter PL decay times (e.g., 3% QY NPLs shows about 0.6 μs PL lifetime), which is comparable to core-type NPLs and QDs. In summary, we attribute the prolonged PL decay times with both reduction in non-radiative recombination pathways and overall thickness increase of the NPLs.

### 3.2. Photoresponsivity Measurement

To obtain a conductive film of nanoparticles, it is required to substitute long-chain surface ligands with short ones. Typically, the surface ligands can govern the HOMO LUMO energy levels of NC, their conductivity, and electron- or hole-doping. For lead chalcogenide NC this task has been solved with numerous different approaches developed for PbS quantum dot-based solar cell fabrication [27]. Briefly, there are two main approaches to passivate the NC surface: in a solution or in a deposited film. In the former, the short-ligand passivated NC typically possesses less stability and is highly restricted in the solvent choice. In the latter, it is much easier to deposit a film from a vast variety of solvents but the ligand exchange process leads to film rearrangement because of appearing cracks, pinholes, and other large-scale defects.

To fabricate conductive films, we adopt the best-performing EDT and TBAI deposited film passivation methods [28]. First, the prepared PbSe/PbS NPLs colloidal solution was precipitated using the excess of isopropanol and centrifugation at 4000 RPM. The supernatant was discarded and the precipitate was redispersed in octane. The NPLs in octane solution were filtered through a 0.22 µm PTFE syringe filter before film deposition. Interdigitated substrates (Ossila S161 and S181) were pre-cleaned and treated with ozone for better adhesion. For film fabrication, NPLs solution was layer-by-layer spin-coated onto a interdigitated substrate at 2000 RPM. After each NPL layer deposition, the film was treated with a solution of either TBAI (10 mg/mL in methanol) or EDT (0.2% vol. in acetonitrile) for 30 s. After the treatment, the spin-coater was turned on to remove the TBAI/EDT solution and rinsed with acetonitrile in both cases. It is worth mentioning that before the TBAI treatment NPL film was rinsed with methylacetate to remove excess unbound oleic acid from the film surface. Ligand exchange efficiency was confirmed via FTIR measurements (Figure S6) by monitoring the CH_2_ bond absorption (2922 cm^−1^ and 2852 cm^−1^), prevalent in the native oleic acid ligands. Additionally, the XPS measurements confirm the iodine presence for the TBAI sample (Figure S4). Both TBAI and EDT treated films demonstrate a uniform surface with a roughness of 17 ± 1 nm (Figure S7).

**Figure 3:**
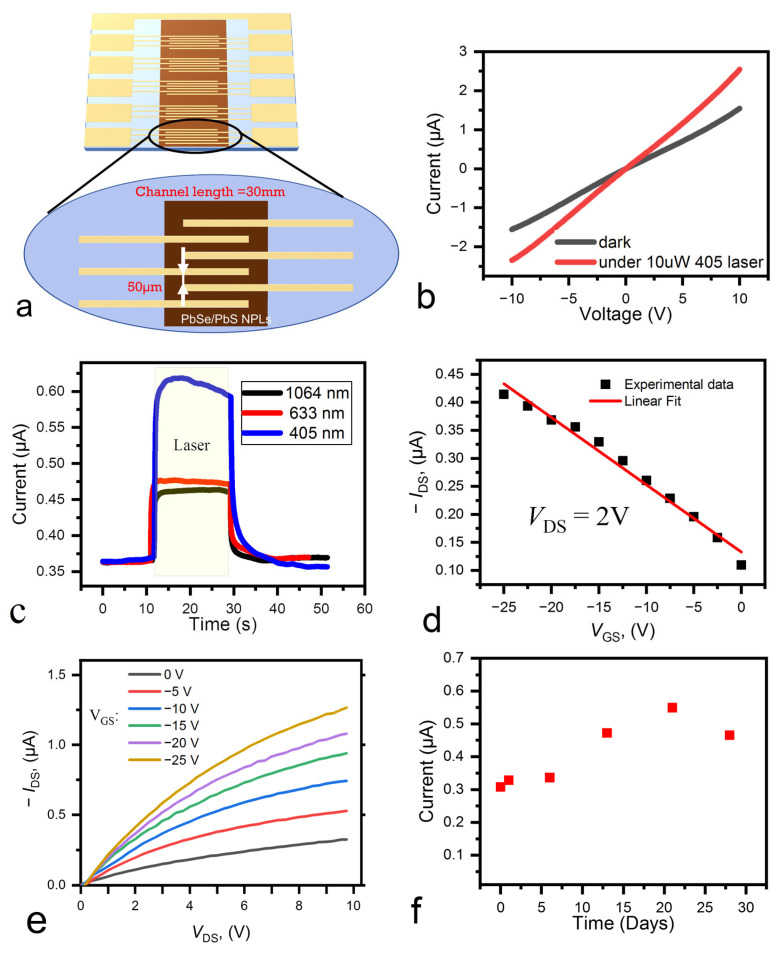
(**a**)—schematic visualization of the studied photoconductor structure; (**b**)—I–V curve of TBAI-treated photoconductor in darkness (grey) and under 405 nm laser irradiation (red); (**c**)—photoresponse test under different laser excitation; (**d**)—current values from (**e**) plotted vs gate voltage and fitted by linear function; (**e**)—series of I–V curves of the FET-device obtained under various gate voltage; (**f**)—current at 2V measured in long-term stability test.

The photoconductor structure was fabricated by depositing the 210 ± 20 nm thick NPLs film onto the interdigitated substrate with a 50 μm channel length and 3 cm channel width (Figure 3a). The EDT-treated films show nonlinear I–V characteristics, probably caused by energy barrier appearance in the device, while TBAI-treated films show more than one order better conductance and a more linear I-V curve (Figure S8 and Figure 3b). Further experiments were conducted on the TBAI-treated films with resistivity of about 2.15 × 10^4^ Ohm·cm. Note, that annealing is typically required for highly efficient films preparation [29]. However, annealing can heavily modify the film itself and the optimization of this process lies beyond the scope of this article.

Responsivity was measured under laser irradiation using a set of wavelengths (405 nm, 633 nm, and 1064 nm) with a power density of 10 µWcm^−1^. The device I–V curve remains linear under laser irradiation (Figure 3b). The device shows responsivity of 1.3 AW^−1^ under 405 nm excitation, 0.74 AW^−1^ under 633 nm excitation, and 0.66 AW^−1^ under 1064 nm excitation, measured at 2V bias (Figure 3c). The dependance of the responsivity on the applied voltage was found to be linear, with better responsivity at positive voltage bias (see Figure S9). This is most probably caused by the p-type conductivity of the obtained films, which will be discussed below.

We also built a field effect transistor (FET) to measure the charge carrier mobility using the methodology proposed by Parfenov et al. [30]. Typically, nanocrystal films are hard to measure in the FET architecture due to film charging during measurements [31]. To prevent film charging, we used the rapid measurement of I–V curves at different gate voltages (Figure 3e). The structure was fabricated analogously to photoconductors using the bottom gate architecture with a silicon/silicon oxide substrate and gold contacts with 30 μm spacing. The dependance of the channel current on the gate voltage was derived from the analysis of the I–V curves (Figure 3d). For the charge mobility calculations, we used a well-known equation [32]:

(1) $$\mu_{lin}=\frac{L}{WC_{ox}V_{ds}}\;\frac{\partial I_{ds}}{\partial V_{gs}},$$

where *W*—channel width, *L*—channel length, *C_ox_*—oxide capacitance, *I_ds_*—channel current, *V_ds_*—voltage between the drain and the source, *V_gs_*—gate voltage. In our experiment we used Ossila S181 substrates with a 30 µm channel length, 1 mm channel width, and SiO_2_ insulating with $C_{ox}$ = 1.09 × 10^−8^ F∙cm^−2^. The samples possess p-type conductivity with carrier mobility of 1.26 × 10^−2^ cm^2^ V^−1^ s^−1^. Additionally, we measured the current growth and decay dynamics using a current amplifier with an oscilloscope (Figure S10). Rise time was found to be <20 ns, while fall time is ~26 µs. The calculated value of device bandwidth is about f_3dB_ = 13 kHz. It is worth noting that our PbSe/PbS NPLs-based device possesses fast response of the optical signal, compared with the existing analogues.

We would like to benchmark our device against known analogues. For the PbSe NPLs there is only one known published result, with responsivity about 50 times less than our device and a cutoff frequency of 10 kHz [16]. It would also be beneficial to compare the developed device with well-studied PbS-based devices. A photoconductor with 1 μm spacing and Cu doped PbS nanosheets as active material showed responsivity of 1739 AW^−1^ for 808 nm excitation, however, the device shows high dark current and neither the bandwidth nor cutoff frequency were stated [33]. Another PbS QD-based device shows responsivity of 2700 AW^−1^ with 18 Hz bandwidth using substrates with 5 μm spacing [9]. The iodide-passivated PbSe QDs photoconductor fabricated on a substrate with 5 μm contacts spacing shows responsivity of 0.71 AW^−1^ for 1400 nm excitation with unclear fall time within 1 s [34]. The charge carrier mobility data are close to PbS NPLs-based FET [35] and exceed iodide-passivated PbSe QDs by about 1 order of magnitude [34]. Note that typical responsivity values for commercially available NIR detectors are about 1 AW^−1^ (Thorlabs, Newton, NJ, USA) and area ranges from 0.08 to 1 mm.

The efficiency of the photoconductor device could depend nonlinearly on the contacts spacing. This is caused by several reasons, such as lower electric field in the active layer, worse material arrangement in larger substrates, or higher trap density in the channel [36,37,38,39]. Thus, our device achieves comparable results, even using the architecture with a relatively high contact spacing of 50 μm.

To test the sample stability, it was stored for 28 days under ambient conditions (20 °C, exposed to light, 60 ± 10% humidity). We found that the sample increases its conductance during long-term storage. We suppose that at least two possible factors might result in this behavior: moisture penetrating the film, and oxidation of the surface. The moisture effect was mitigated by putting the photoconductor into the vacuum for about 3 h before the measurement. Even with vacuum treatment we observe the increase in the sample conductivity during storage time (Figure 3f). Similar after-storage conductivity increase has been observed before for various lead chalcogenide-based devices due to the initial p-doping, due to the slight oxidation [28,40]. The conductivity starts to decrease on the 28th day probably due to excessive oxidation. However, such a result is promising for NC-based materials and overperforms typical PbSe degradation rates [34].

To summarize, our device shows lower responsivity than those reported for PbS QDs-based, PbSe QDs-based, and PbS NPLs analogues, but is still compatible with commercially available analogs. The clear advantage over the compared devices is the large contact spacing (50 μm) that proves our device has better scalability, which is beneficial for further development and commercialization. Moreover, our device shows the best bandwidth value among those reported for lead chalcogenide-based devices with similar architecture.

## 4. Conclusions

In this paper we have reported on the novel synthetic routine for PbSe/PbS core/shell NPLs synthesis via cation exchange. The exchange from CdSe/CdS to PbSe/PbS was monitored with optical spectroscopy and shows the slow exchange reaction with continuously red shifting PL band. The shell growth enhances the NPLs environmental stability, both in films and in colloidal solution.

We have fabricated photoconductor devices from PbSe/PbS core/shell NPLs using a convenient structure and well-studied methods. The resulting device shows record responsivity for PbSe NPLs with fast optical response and a rather high 13 kHz bandwidth. The overall film shows a relatively good resistivity (2.15 × 10^4^ Ohm·cm), high carrier mobility (1.26 × 10^−2^ cm^2^ V^−1^ s^−1^), and responsivity of 0.66 AW^−1^ in the NIR region.

Based on the quality of the photoresponse and high bandwidth, we demonstrate that PbSe/PbS NPLs-based devices have the potential to be applied in the field of photodetection. However, there are still a few milestones to reach. First, thermal annealing for the NPLs films should be studied. As we showed in the stability test, the device properties evolve during storage. Annealing could force slow evolution of the device properties to a higher performance state where encapsulation quenches further degradation. Second, a direct synthesis routine should be designed for commercialization of such devices. The oriented attachment procedure has been previously reported for PbS NPLs and could be tested for PbSe NPLs production.

## Data Availability

The data that support the findings of this study are available from the corresponding author upon reasonable request.

## Supplementary Materials

The following supporting information can be downloaded at: https://www.mdpi.com/article/10.3390/nano13233051/s1, Figure S1: The absorption spectra of the aliquots depicted in Figure 1; Figure S2: EDX graph of PbSe/PbS NPLs; Figure S3: XPS spectra of as-synthesized PbSe/PbS NPLs; Figure S4: XPS spectra of TBAI-treated PbSe/PbS NPLs; Figure S5: The PL spectra of the PbSe/PbS NPLs before (black) and after(red) 21 days of storage in ambient air conditions and room temperature; Figure S6: FTIR Spectra of PbSe/PbS films with different ligand shell. Black—oleic acid shell, red—after EDT treatment, green—after TBAI treatment; Figure S7: AFM (a) and SEM (b) images of the TBAI film; Figure S8: I-V curves of PbSe/PbS core-shell NPLs layers treated with EDT without(gray) and under 10 µWcm^−1^ 405 nm laser excitation(red); Figure S9: The dependence of responsivity on applied voltage, measured under 405 nm irradiation at 10 µWcm^−1^ power density; Figure S10. The current of the PbSe/PbS NPLs-based photoconductor sample under fast 532nm laser pulses used for fall and rise times measurements; Table S1: Elemental composition of the PbSe/PbS NPLs calculated from EDX data; Table S2: PbSe/PbS NPLs atomic percentages from the XPS measurement. Values given for films with as-synthesized OA ligands and after TBAI treatment.
