# Supplementary material for: PbSe/PbS Core/Shell Nanoplatelets with Enhanced Stability and Photoelectric Properties
Source: Nanomaterials (Basel). 2023 Nov 29;13(23):3051. doi: 10.3390/nano13233051 (PMC10707907; doi:10.3390/nano13233051)
Supplement: Supplementary file 1 [file nanomaterials-13-03051-s001.zip › nanomaterials-2718459-supplementary.pdf]

Supplementary materials

PbSe/PbS core/shell nanoplatelets with enhanced stability and photoelectric properties

Anton A. Babaev*[1], Ivan D. Skurlov[1], Sergei A. Cherevkov[1], Peter S. Parfenov[1], Mikhail A Baranov[1], Natalya K. Kuzmenko[2], Aleksandra V. Koroleva [3], Evgeniy V. Zhizhin[3] and Anatoly V. Fedorov[1]

1 PhysNano Department, ITMO University, Saint Petersburg 197101, Russia
2 Research Center for Optical Materials Science, ITMO University, Saint Petersburg 197101, Russia
3 St. Petersburg State University, St. Petersburg, 199034, Russia
* Correspondence: a.a.babaev1@gmail.com

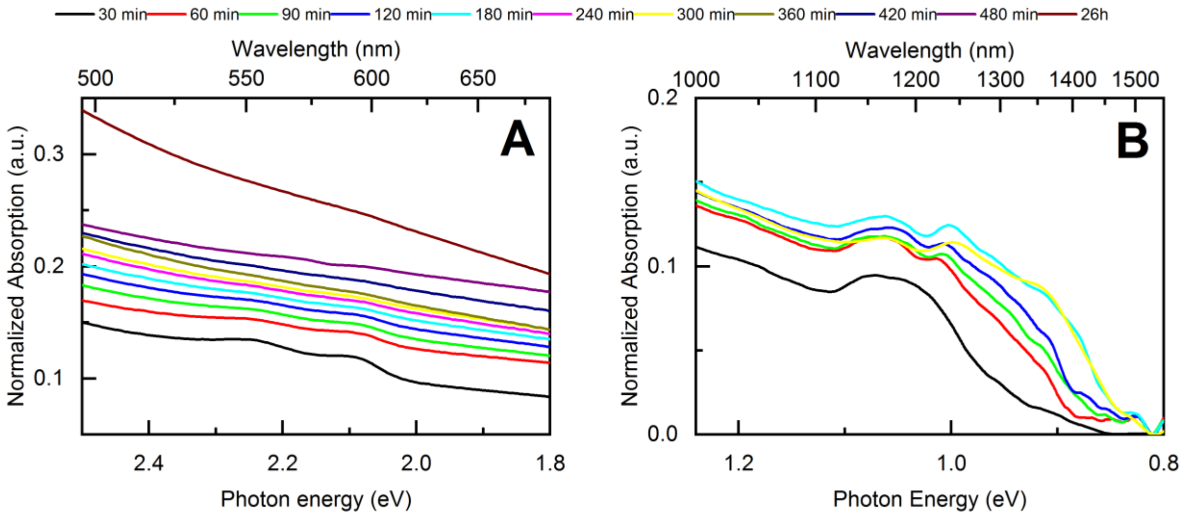

Figure S1. The absorption spectra of the aliquots depicted in Figure 1

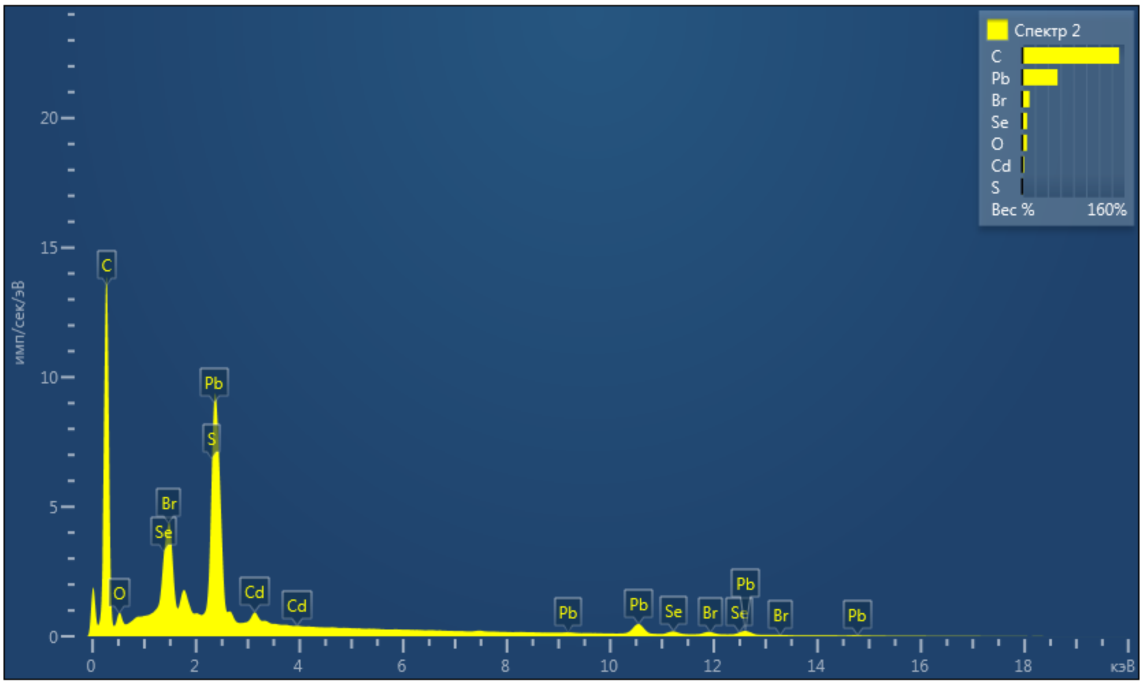

Figure S2. EDX graph of PbSe/PbS NPLs

Table S1. Elemental composition of the PbSe/PbS NPLs calculated from EDX data

Element	C	O	S	Se	Br	Cd	Pb	Sum:
Atom percentage (%)	92.27	3.49	0.31	0.72	1.07	0.19	1.96	100

We note that Cd/Pb rate is different for OA-capped and TBAI-treated films with values of 0.155 and 0.187, respectively. We speculate the additional $PbBr_2$ z-type ligand could be removed by resulting in Cd/Pb change. The overall difference of PbCd/SSe rate of 1.52 and 1.42 for OA-capped and TBAI-treated films are also support this statement.

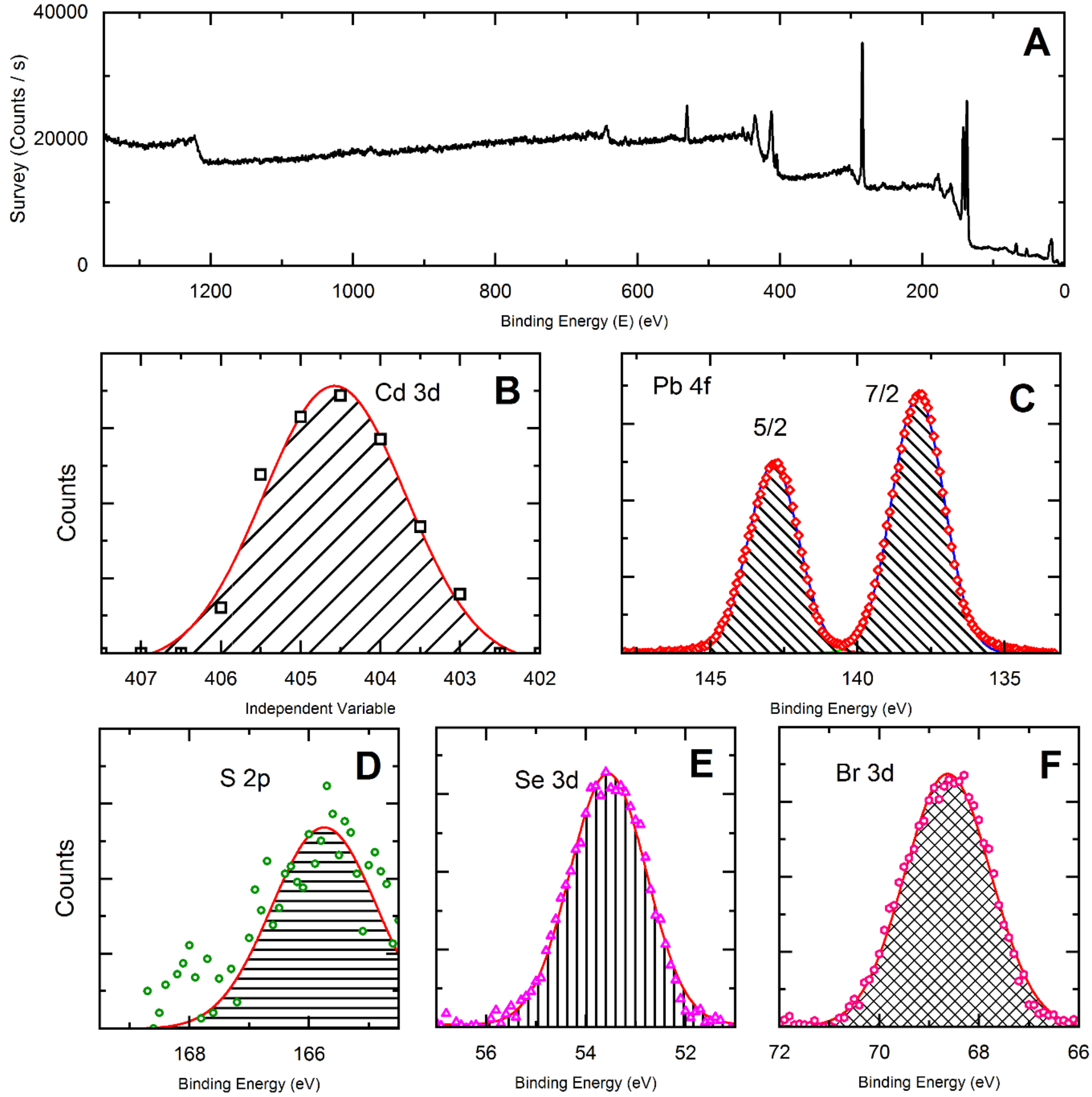

Figure S3. XPS spectra of as-synthesized PbSe/PbS NPLs. A – survey spectra, B – Cd 3d, C – Pb 4f, D -S 2p, E – Se 3d, F – Br 3d.

Table S2. PbSe/PbS NPLs atomic percentages from the XPS measurement. Values given for films with as-synthesised OA ligands and after TBAI treatment

Ligand shell	C	O	S	Se	Br	Cd	Pb	I
OA	80.54	9.3	0.75	2.48	2.01	0.66	4.26	0
TBAI	44.81	4.51	3.67	12.58	3.21	3.66	19.55	8.01

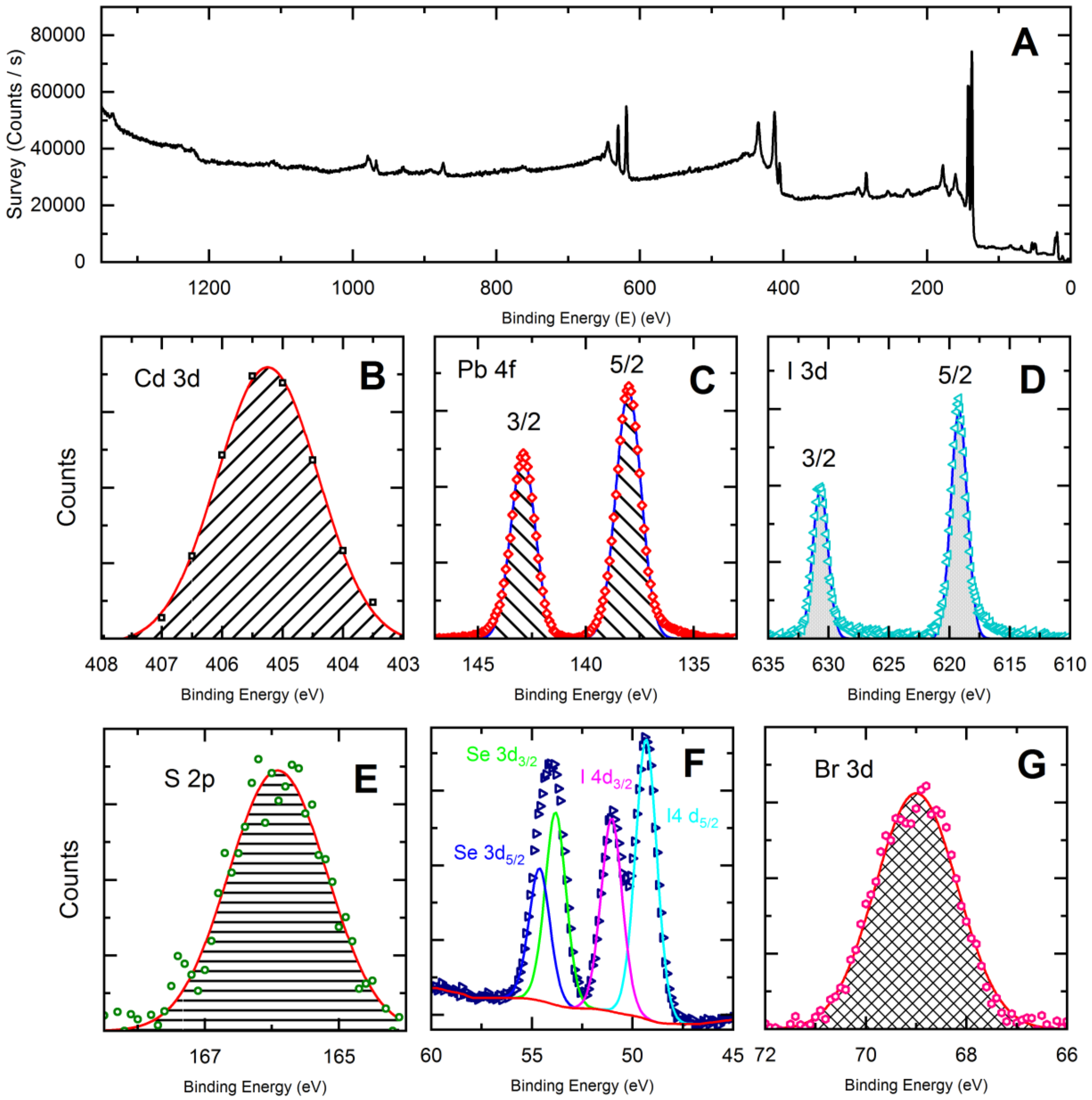

Figure S4.XPS spectra of TBAI-treated PbSe/PbS NPLs. A – survey spectra, B – Cd 3d, C – Pb 4f, D – I 3d, E – S 2p, F – Se 3d and I 4d, G – Br 3d.

Stability in solution

The sample of NPLs in octane was stored in ambient air conditions and room temperature. The PL spectra was recorded in similar conditions. Figure S3 shows the recorded normalized PL spectra.

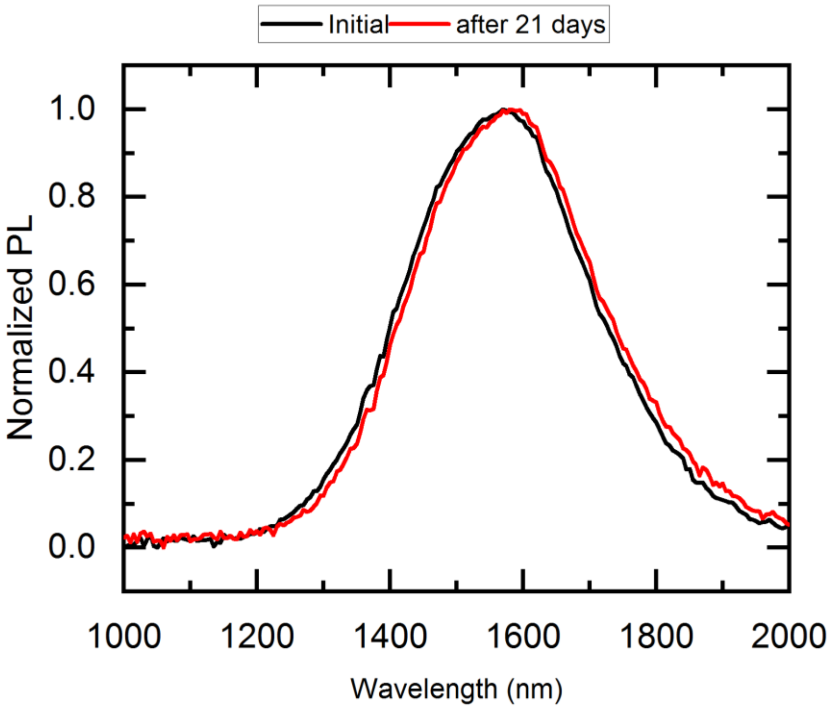

Figure S5. The PL spectra of the PbSe/PbS NPLs before (black) and after(red) 21 days of storage in ambient air conditions and room temperature

Ligand exchange efficiency was monitored by the presence of CH_2 bond vibration prevalent int the oleic acid. These can be seen in in the FTIR transmittance spectra at 2922 cm^{-1} and 2852 cm^{-1}. Figure S4 shows that treatment with either TBAI or EDT greatly reduces the intensity of such vibrations. Thus we can assume that oleic acid was mostly replaced by either I^- ions or EDT molecules.

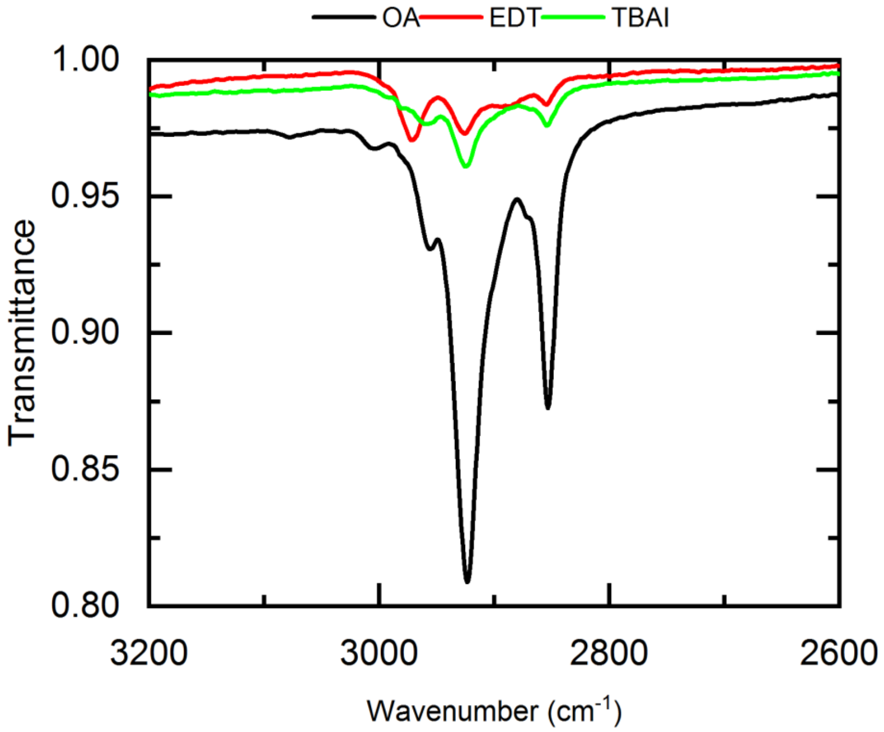

Figure S6. FTIR Spectra of PbSe/PbS films with different ligand shell. Black – oleic acid shell, red – after EDT treatment, green – after TBAI treatment.

Representative SEM and AFM images of TBAI film are shown in Figure S5.

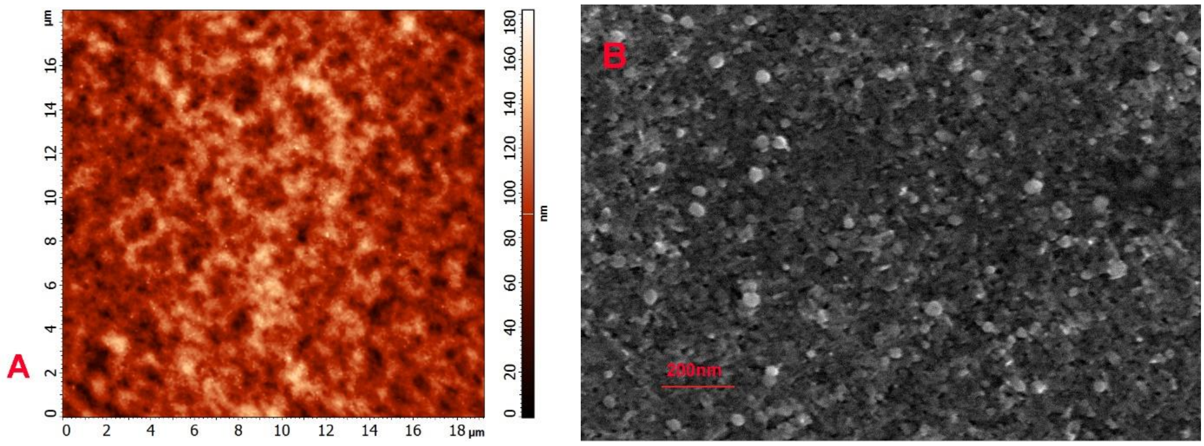

Figure S7. AFM (a) and SEM (b) images of the TBAI film

Responsivity calculations

Responsivity was calculated using commonly used equation:

$$R = \frac{I_{light} - I_{dark}}{P_{light}}$$

where I_{dark} – dark current, I_{light} – current under illumination of 10µW·cm^{-2} light, P_{light} – power of incident light calculated as product of light source power density and structure area.

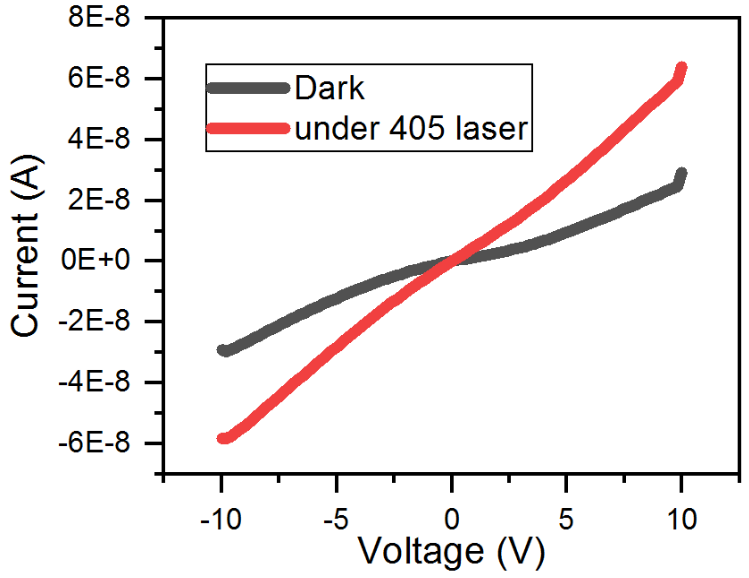

Figure S8. I-V curves of PbSe/PbS core-shell NPLs layers treated with EDT without(gray) and under 10 μWcm^{-1} 405 nm laser excitation(red).

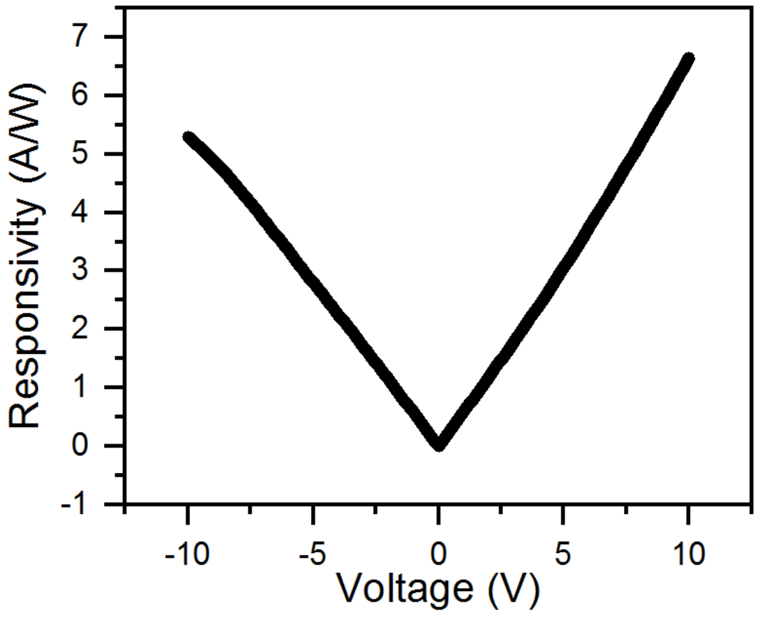

Figure S9. The dependence of responsivity on applied voltage, measured under 405 nm irradiation at 10 μWcm^{-1} power density.

Bandwidth measurements

The bandwidth of the photoconductor structure was calculated from the well-known relation

$$\tau_r \cong \frac{0.35}{f_{3dB}}$$

For the rise and fall time measurement we apply tektronix tds 2022b oscilloscope combined with HCA-10M-100K-C high-speed current amplifier. The rise time was determined as the time difference between 10% and 90% current as depicted in Figure S6.

The fall time was determined fitted using 3-exponentical decay curve and the curve was used for the 10% and 90% current offset determination as depicted in Figure S6.

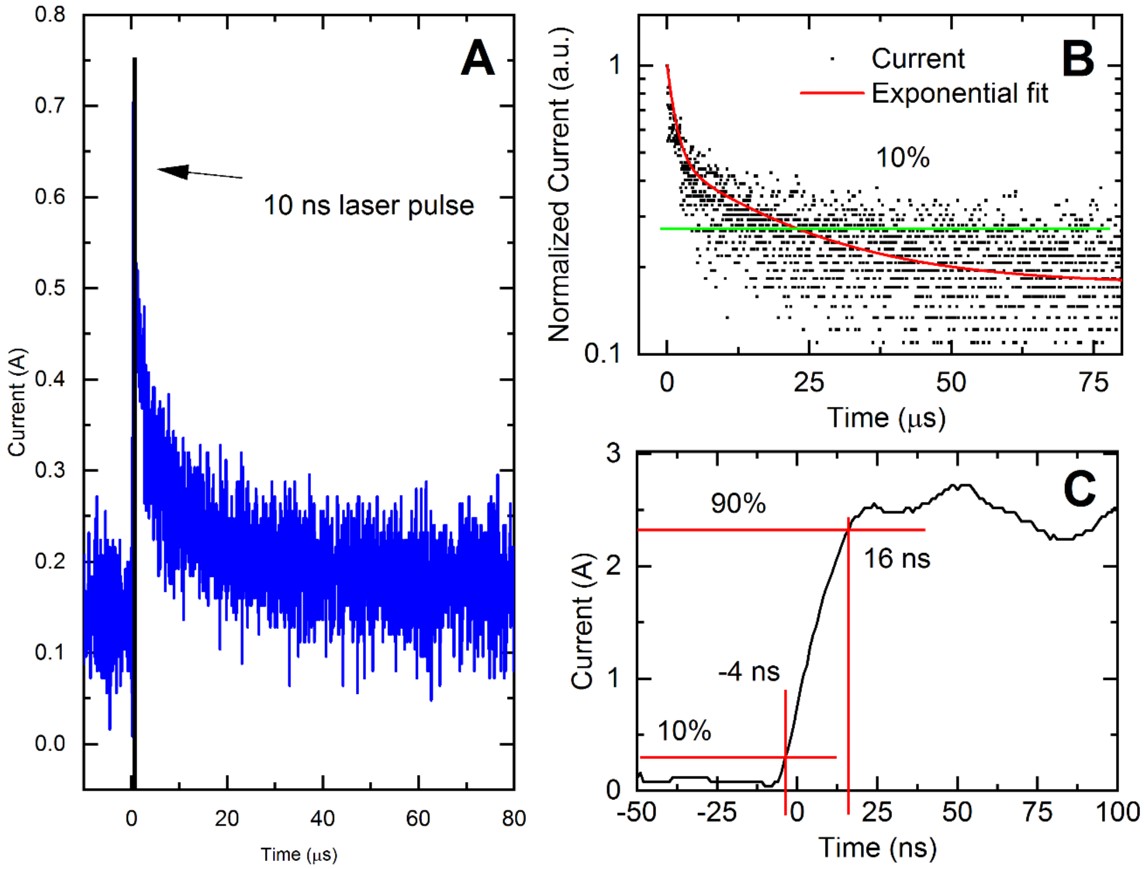

Figure S10. The current of the PbSe/PbS NPLs-based photoconductor sample under fast 532nm laser pulses used for fall and rise times measurements.
